# Sialoadhesin (CD169) Expression in CD14+ Cells Is Upregulated Early after HIV-1 Infection and Increases during Disease Progression

**DOI:** 10.1371/journal.pone.0000257

**Published:** 2007-02-28

**Authors:** Antoinette C. van der Kuyl, Remco van den Burg, Fokla Zorgdrager, Fedde Groot, Ben Berkhout, Marion Cornelissen

**Affiliations:** Laboratory of Experimental Virology, Department of Medical Microbiology, Centre for Infection and Immunity Amsterdam (CINIMA), Academic Medical Centre of the University of Amsterdam, Amsterdam, The Netherlands; Institut Pasteur Korea, Republic of Korea

## Abstract

**Background:**

Sialoadhesin (CD169, siglec-1 or Sn) is an activation marker seen on macrophages in chronic inflammatory diseases and in tumours, and on subsets of tissue macrophages. CD169 is highly expressed by macrophages present in AIDS-related Kaposi's sarcoma lesions. It is also increased on blood monocytes of HIV-1 infected patients with a high viral load despite antiretroviral treatment.

**Methodology/Principal Findings:**

We investigated expression of sialoadhesin in untreated HIV-1 and HHV-8 infected patients, by real-time PCR and FACS analysis to establish its expression in relation to infection and disease progression. Patients analysed were either HIV-1 seroconverters (n = 7), in the chronic phase of HIV-1 infection (n = 21), or in the AIDS stage (n = 58). Controls were HHV-8 infected, but otherwise healthy individuals (n = 20), and uninfected men having sex with men (n = 24). Sialoadhesin mRNA was significantly elevated after HIV-1, but not HHV-8 infection, and a further increase was seen in AIDS patients. Samples obtained around HIV-1 seroconversion indicated that sialoadhesin levels go up early in infection. FACS analysis of PBMCs showed that sialoadhesin protein was expressed at high levels by approximately 90% of CD14^+^ and CD14^+^CD16^+^cells of HIV-1^+^ patients with a concomitant 10-fold increase in sialoadhesin protein/cell compared with uninfected controls.

**Conclusions/Significance:**

We have shown that sialoadhesin is induced to high levels on CD14^+^ cells early after HIV-1 infection in vivo. The phenotype of the cells is maintained during disease progression, suggesting that it could serve as a marker for infection and probably contributes to the severe dysregulation of the immune system seen in AIDS.

## Introduction

HIV-1 infection of humans is traditionally divided into three stages: the acute phase lasting several weeks, when viral RNA levels are high and specific antibodies are low, followed by a relatively long period of chronic infection starting at the moment of seroconversion (when HIV-specific antibody tests become positive). Finally, the combined effects of the acute and chronic disease stage results in a total collapse of the immune system resulting in multiple opportunistic infections: the AIDS phase. HIV-1 infection is characterized by the expression of large amounts of proinflammatory cytokines, which are involved in immune suppression by promoting both CD4^+^ and CD8^+^ T cell apoptosis (for a review: see [1]). The cytokine signalling systems are further deregulated by virally encoded proteins such as Nef, Tat and Vpr. These chronic inflammatory conditions are linked to elevated virus production and immune dysfunction.

Applying Serial Analysis of Gene Expression (SAGE) to AIDS-related Kaposi's sarcoma (AIDS-KS) tissue and comparing its expression profile to that of normal skin, revealed several transcripts that were upregulated in AIDS-KS [2]. Of particular interest was the sialic acid binding receptor sialoadhesin (CD169, siglec-1 or Sn), which is seen on activated macrophages in chronic inflammation and in tumours, e.g. on macrophages found in multiple sclerosis, atherosclerosis, and rheumatoid arthritis [3], on monocytes from HIV-1 infected patients treated with antiretroviral therapy, especially those with a high viral load [4], and on dendritic cells infected with human rhinovirus strain 14 [5]. Sialoadhesin mediates cell-cell interactions by recognizing Neu5Acα2,3Gal in either N- or O-glycans on the surface of several leukocyte subsets [3], [6]. To evaluate expression of sialoadhesin during different stages of HIV-1 infection, we investigated sialoadhesin mRNA levels by real-time PCR in peripheral blood mononuclear cells (PBMCs) collected from patients participating in the Amsterdam Cohort Studies on HIV/AIDS. The expression level of sialoadhesin in AIDS-KS was compared with four control groups consisting of a-symptomatic HIV-1 infected patients, HHV-8 infected patients, patients with AIDS other than AIDS-KS, and non-infected controls belonging to the risk group (men having sex with men). Sialoadhesin mRNA levels were also compared in PBMCs from an additional set of patients sampled before and after the moment of seroconversion for HIV-1 (seroconverters). FACS analysis was used to investigate whether the upregulation of sialoadhesin represents an increased expression per cell, or an increase in cells expressing sialoadhesin. In asymptomatic HIV-1 infected patients, sialoadhesin protein expression was increased in the total PBMC fraction, including high levels on approximately 90% of both CD14^+^ and CD14^+^CD16^+^cells, with a mean two-fold expansion of the latter cell type.

## Results

### Expression of sialoadhesin mRNA

**Figure 1:**
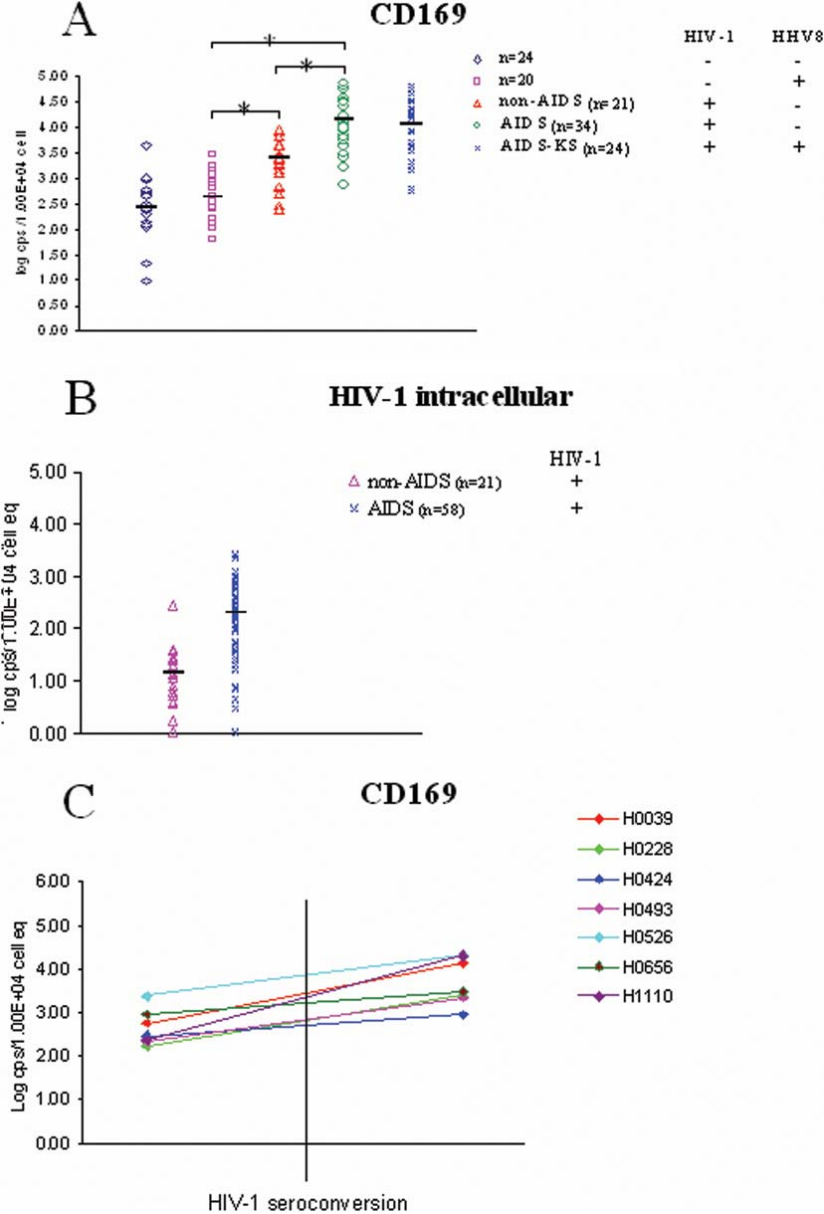
Real-time PCR analysis of sialoadhesin mRNA levels in PBMCs of HIV-1 infected patients and controls. A. Real-time PCR analysis of sialoadhesin mRNA levels in PBMCs derived from five sets of patients with or without HIV-1 infection at different stages of disease. Patients with AIDS but not AIDS-KS, and patients with AIDS-KS were analysed separately. Levels were significantly different (p≤0.0005) between uninfected controls/HHV-8 infected patients, and HIV-1 infected patients, respectively. Levels were also significantly different (p≤0.0005) between HIV-1 infected patients and AIDS patients. Significant differences are indicated with an asterisk. No significant differences were found between patient groups not infected with HIV-1 (p = 0.1), or between AIDS and AIDS-KS patients (p = 0.14). B. Real-time PCR analysis of HIV-1 mRNA levels in PBMCs derived from either HIV-1 infected asymptomatic patients or patients with AIDS (including AIDS-KS). The elevated HIV-1 load in AIDS patients compared to asymptomatic HIV-1^+^ patients is highly significant (p≤0.0001). C. Real-time PCR analysis of sialoadhesin mRNA levels in PBMCs obtained from eight patients before and after HIV-1 seroconversion. Sialoadhesin mRNA was analysed in the first HIV-1 seropositive sample of each patient, which is within three months from the real seroconversion event in the Amsterdam Cohort Studies on HIV/AIDS, as samples are taken on a three-monthly basis.

Sialoadhesin mRNA was quantified in PBMCs of patients infected with HIV-1 and/or HHV-8 (Fig. 1A). A highly significant difference (p≤0.0005) in sialoadhesin mRNA levels was seen in AIDS patients (both AIDS-KS and non-AIDS-KS, median copy numbers per group ranged from 4.83×10^3^–2.35×10^4^/10^4^ PBMC), and in HIV-1 infected a-symptomatic patients (median = 2.49×10^3^ copies/10^4^ PBMC) when compared with non-infected risk-group patients (median = 2.69×10^2^ copies/10^4^ PBMC) or with patients with exclusively an HHV-8 infection (median = 4.28×10^2^ copies/10^4^ PBMC). Sialoadhesin levels between AIDS-KS and other AIDS patients did not differ significantly (Fig. 1A). These findings suggest that sialoadhesin mRNA is not specifically increased in AIDS-KS, as suggested by the earlier SAGE analysis, but is upregulated by HIV-1 infection. Differences in sialoadhesin copy number between HIV-1^+^ asymptomatics and AIDS patients, including AIDS-KS patients, were statistically significant, with higher expression in the AIDS group. This further upregulation during HIV pathogenesis could be related to a difference in HIV-1 copy numbers in PMBCs, as these were, not unexpected, found to differ significantly between AIDS and non-AIDS patients (p≤0.0001), with higher numbers in AIDS patients (Fig. 1B). However, sialoadhesin mRNA copy numbers were only moderately correlated with HIV-1 RNA copy numbers (Pearson correlation coefficient = 0.42, p = 0.0002). Overall, there was a trend that in PBMCs high HIV-1 intracellular copy numbers accompanied high sialoadhesin mRNA copy numbers, but this was not so for all patients. Sialoadhesin mRNA copy numbers did not correlate with CD4^+^ cell counts in AIDS patients (Pearson correlation coefficient = −0.125, p = 0.51).

To analyse the effect of HIV-1 infection in more detail, sialoadhesin copy numbers were determined in samples from seven HIV-1 infected patients taken before and after HIV-1 seroconversion (Fig. 1C). The post-seroconversion sample was the first HIV-1 positive sample identified in individuals tested at three monthly intervals, therefore within three months of seroconversion. Indeed, sialoadhesin copy numbers increased from a median of 3.09×10^2^ copies/10^4^ PBMC to 3.00×10^3^ copies/10^4^ PBMC (p = 0.0016) after HIV-1 seroconversion, suggesting either an increase of sialoadhesin mRNA in a certain cell population, the expansion of a cell population expressing sialoadhesin after HIV-1 infection, or a loss of cells with low sialoadhesin expression, or a combination of these.

### Fluorescence-activated cell sorter (FACS) analysis of PBMCs

**Figure 2:**
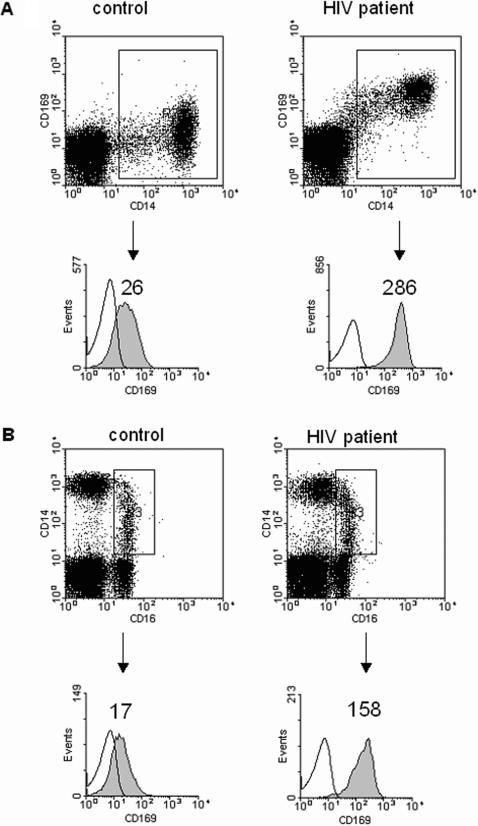
Expression of CD14, CD16, and sialoadhesin on monocytes from an HIV-1 infected and uninfected patient. A. Sialoadhesin (CD169) expression on CD14^+^ cells from a healthy human control (left) and an HIV-1 infected patient (right). The mean fluorescent intensity of CD169 from the CD14^+^ cells increases from 26 in the control cells (left) to 286 in the cells from the HIV-1 infected patient (right). B. CD14^+^CD16^+^ cells were gated and their expression of sialoadhesin is shown in the histograms. The mean fluorescent intensity of CD169 from the CD14^+^CD16^+^ cells increases from 17 in the control cells (left) to 158 in the cells from the HIV-1 infected patient (right). The data are representative of three experiments.

Increase of sialoadhesin expression at the mRNA level as measured by real-time PCR in our cohort could either mean that expression per cell has increased, that the number of sialoadhesin expressing cells has increased, or a combination of both. To differentiate between these possibilities, we analysed CD14^+^ cells and the subset of CD14^+^CD16^+^ cells from three healthy donors and three treatment-naïve, asymptomatic HIV-1 infected patients for sialoadhesin protein expression by flow cytometry (Table 1). A representative example of the FACS analysis is shown in Fig. 2 (panel A: CD14^+^ cells, panel B: CD14^+^CD16^+^ cells). Staining with mAbs for either CD14^+^, CD16^+ ^or CD169^+^ showed that sialoadhesin expression increases dramatically in CD14^+^ cell fractions, both in positive cell numbers as well as in the amount of molecules per cell, in HIV-1 infected patients compared with uninfected controls (Table 1). A median of approximately 90% of CD14^+^ and CD14^+^CD16^+^ cells expressed sialoadhesin at levels that were approximately 10-fold higher than in healthy donors. The number of CD14^+^CD16^+^ cells is increased approximately twofold in the PBMC population of HIV-1 infected patients compared with healthy controls (Table 1, median increase from 2,1% to 4,6%). The total percentage of CD14^+^ cells does not change notably after HIV-1 infection (Table 1, median percentage in healthy donors = 13,6% versus 10,4% in HIV-1 infected patients), nor does the amount of cells not expressing CD14, CD16 and sialoadhesin (66.47 and 67.28%, respectively), or the percentage of cells expressing sialoadhesin, but not CD14 and CD16 (0.21 and 0.42%, respectively).

**Table 1:** Percentage of cells expressing CD14, CD16, and/or sialoadhesin of the total PBMC fraction, as determined by FACS analysis

CD14	CD16	Sialoadhesin	Healthy donors: median % of total (range)	HIV-1^+^ patients: median % of total (range)
**No**	**No**	**No**	66.47(54.79–70.88)	67.28 (63.50–72.85)
**No**	**No**	**Yes**	**0.21 (0.05–0.22)**	**0.42 (0.19–1.72)**
**Yes**	**No**	**No**	12.67 (12.47–13.43)	0.79 (0.31–8.16)
**Yes**	**No**	**Yes**	**0.88 (0.17–2.38)**	**9.65 (4.15–14.69)**
**Yes**	**Yes**	**No**	1.99 (1.92–3.16)	0.56 (0.08–2.57)
**Yes**	**Yes**	**Yes**	**0.11 (0.04–0.35)**	**4.00 (0.90–5.31)**
**No**	**Yes**	**No**	14.67 (10.81–29.18)	10.78 (10.30–20.50)
**No**	**Yes**	**Yes**	**0.01 (0.01–0.06)**	**0.17 (0.03–1.09)**

## Discussion

Sialoadhesin mRNA and protein expression in PBMCs was significantly increased after HIV-1 infection in vivo. An additional raise was seen in AIDS-patients compared to asymptomatic HIV-1 infected patients. An analysis of blood samples taken before and after HIV-1 seroconversion indicated that the initial upregulation of sialoadhesin occurs rapidly after HIV-1 infection, and is maintained during the chronic phase of the infection. Sialoadhesin expression is increased after the moment of seroconversion (when specific antibody tests become positive), which is approximately 3 months after the acute HIV infection. Possibly, sialoadhesin levels already go up during the acute phase of the infection. However, due to the rarity of acute phase samples, it was not feasible in this study to assess if sialoadhesin expression is already increased in this stage of the HIV-1 infection.

In mice, approximately 10^6^ molecules/cell of sialoadhesin are found, making it one of the most highly expressed surface molecules on macrophages [7]. Levels of mRNA and protein correlate well in mice [8]. Similar results were obtained in humans in this study, suggesting that sialoadhesin expression is mainly regulated at the transcriptional level, although it could be that cells with low sialoadhesin expression disappear, or that a novel cell subset expressing sialoadhesin emerges. Subsets of PBMC are generally different in HIV-infected patients compared with non-infected individuals, especially in the AIDS phase, when lymphocytes are significantly decreased and the monocyte fraction is increased. FACS analysis of blood cells obtained from HIV-1 infected individuals with a relatively high viral load showed that sialoadhesin is mainly expressed by and increased on CD14^+^ cells, including CD14^+^CD16^+^ monocytes. The potential influence of differences in the cellular subsets of healthy vs. HIV infected individuals seems limited here as the total amount of CD14^+^ monocytes does not differ appreciably between the two patient groups, although the percentage of CD14^+^CD16^+^ monocytes is larger after HIV-1 infection. However, to draw more firm conclusions more patients should be analysed.

Sialoadhesin mRNA was found in a monocytic cell line, a promonocytic cell line, cells of the myeloerythroid lineage, but not in B-cells [3]. Expression of the protein was detected in tissue macrophages, but not in blood monocytes from healthy donors [3]. However, sialoadhesin was present on monocytes from treated, chronically HIV-1 infected patients with high viral loads [4], leading to the suggestion that these patients harbour a circulating CD14^+^ monocyte/macrophage hybrid phenotype with chronic inflammatory characteristics.

Two monocyte subsets are currently recognized in peripheral blood, characterized by their level of CD14 and CD16 expression (CD14^high^CD16^−^, and CD14^low^CD16^+^, representing approximately 90% and 10% of blood monocytes, respectively [9]). An expansion of CD14^+^CD16^+^ monocytes has been observed after HIV-1 infection [10]–[12] and other inflammatory conditions, while numbers of CD14^++^ monocytes do either not change [11], [13], or decrease slightly [10], in agreement with our observations. It has been assumed that CD14^+^CD16^+^ monocytes are a distinct “proinflammatory” subset of the monocyte lineage [14]. However, as CD14^+^CD16^+^ monocytes exhibit features of mature tissue macrophages (such as downregulation of CD11b, CD14, and CD33 expression, and upregulation of CD16 expression), it is also possible that they represent either an activated or a predifferentiated form of monocyte. In vitro experiments suggested the former, as inflammatory cytokines were able to induce a CD14^+^/CD16^+^- phenotype in cultured monocytes [15]. An increasing proportion of monocytes in HIV-1 infected patients show an activated, mature phenotype during disease progression when tested for markers such as CD11b, HLA-DR, CD45, CD16, TNF-alpha, PGE2, and IL-6 [13]. Furthermore, expression of several other molecules is increased on monocytes after HIV-1 infection, e.g. CD23 [16], CD36 [17], CD14 [11], CD126 [18], HLA-G [19], and sialoadhesin ([4] and this study). The CD169^+^ phenotype arises shortly after HIV-1 seroconversion, is maintained during progression to AIDS, and does not specifically occur during therapy failure, as suggested earlier [4].

Human sialoadhesin is highly upregulated in certain (inflammatory) diseases such as multiple sclerosis, atherosclerosis, and rheumatoid arthritis, in breast cancer tumour-infiltrating macrophages [20], [21], and by HIV-1 infection ([4], and this study). The function of sialoadhesin is still not completely clear [20]. Sialoadhesin mediates cell-cell interactions by recognizing Neu5Acα2,3Gal in either N- or O-glycans on the surface of several leukocyte subsets [3], [6], suggesting that sialoadhesin could be involved in adhesion processes. Monocytes (and also lymphocytes) show increased adhesion to endothelial cells after HIV infection, leading to an increase in cardiovascular disease, atherosclerosis and Kaposi's sarcoma in HIV-positive patients (reviewed in [22]). Increased expression of sialoadhesin could be related to this phenomenon. On the other hand, dendritic cells express sialoadhesin after infection with human rhinovirus 14, and here it was shown to function as an inhibitory receptor involved in inducing anergy in T-cells [5]. CD33-related members of the siglec family (subgroup 2) have been shown to have inhibitory functions in the immune system [23]. It is likely that the distinct subgroup 1 siglecs (siglec-1, -2, and –4) have similar, negative regulatory functions in the immune system. Elevated, enduring expression of sialoadhesin in chronic inflammatory conditions (including HIV infection) and certain types of cancer, probably induced by high-level cytokine expression, then has a lasting negative, inhibitory effect on the immune system that finally results in opportunistic infections and/or the occurrence of tumours due to immune system malfunction. Chronic inflammation is a risk factor for many types of cancer (for a review, see [24]), and is characterized by immune system unresponsiveness (see e.g. [25]).

Only higher animals and a few microorganisms can synthesize sialic acids, but some pathogens have found ways to capture these sugars from their hosts (reviewed in [26]), possibly to provide an immunologic disguise. In pigs, siglec-1 can act as an endocytic receptor for Porcine Reproductive and Respiratory Syndrome Virus (PRRSV), a single-stranded RNA virus [27], whereby sialic acids on the virus particle are essential for infection of porcine macrophages [28]. The HIV-1 envelope protein gp120 is highly glycosylated and sialylated [29], [30], suggesting that the presence of sialic acids on the virion surface influences host production of sialoadhesin.

Concluding, we have shown that early after HIV-1 infection, sialoadhesin is induced to high levels on CD14^+^ cells of the blood. The phenotype of the cells is maintained during disease progression, suggesting that it could serve as a marker for infection and probably contributes to the severe dysregulation of the immune system seen in AIDS.

## Materials and Methods

### Patient characteristics

Blood samples were collected from participants of the Amsterdam Cohort Studies on HIV/AIDS (www.amsterdamcohortstudies.org). PBMCs isolated from a total of 131 individuals, all Caucasian homosexual males, were selected for this study. They were divided into the following groups: 24 AIDS-KS patients, 34 AIDS patients, 20 HHV-8 infected patients, 21 HIV-1 infected a-symptomatic patients, 7 HIV-1 seroconverters, and 24 non-infected controls belonging to the risk group (men having sex with men). Of the AIDS-KS patients, 13 ( = 54%) had quantifiable HHV-8 DNA in their PBMCs [31]. Of the non-KS AIDS patients, 5 ( = 15%) had quantifiable HHV-8 DNA in their PBMCs, and 15 (44%) were seropositive for HHV-8 in either an ORF65 and/or an ORF73 ELISA [32]. AIDS-KS patients have been analysed separately from other AIDS patients, as high expression of sialoadhesin in AIDS-KS lesions [2] suggested that levels of sialoadhesin could be different in this patient group.

### RNA and DNA extraction

Nucleic acids were isolated from 2.5×10^5^ PBMCs using silica and guanidium thiocyanate [33] in a final elution volume of 62.5 µl. RNA and DNA can be isolated simultaneously by this method.

### Real-time quantitative PCR assay

Copy numbers of sialoadhesin were determined with TaqMan assays using primers (upstream primer: 5′CACCTCCAAGTGAAGTATGCCC 3′, downstream primer: 5′ CCTGGAAGG ATGTTCCTCCC 3′, probe: 5′AAGGGTGTGAAGATCCTCCTCAGCCC 3′) designed with Primer Express software (ABI, Foster City, CA, USA). First, 2.5 µl isolated RNA was converted into cDNA following the instructions in the ABI Taqman Reverse Transcription reagents kit (ABI, Foster City, CA, USA). Each RT reaction contained 1 µl 10×RT buffer, 2.2 µl MgCl_2_ (25 mM), 2 µl dNTPs (10 mM) 1.5 µl random hexamers (50 µM), 0.2 RNase inhibitor (20 U/µl), 0.35 µl multiscribe RT (50 U/µl), 0.35 µl H_2_O and 2.5 µl sample eluate ( = 10,000 cell equivalents). The RT-reaction started with 10 min. 25°C, 30 min. 48°C and 5 min. 95°C. Subsequent PCR reactions were done according to Platinum® Quantitative PCR Supermix UDG (Invitrogen Life Technologies, Carlsbad, USA). Each reaction contained 10 µl RT-reaction and 40 µl of PCR mixture consisting of 25 µl supermix, 3.6 mM MgCl_2_, 0.9 µM forward primer and reverse primer, 0.2 µM Taqman probe and 1 µl ROX reference dye (50× concentration). HIV-1 copy numbers in PBMCs were measured with a gag-specific Taqman assay (upstream primer: 5′ AAAGAGACCATCAATGAGGAAGCT 3′, downstream primer: 5′ TCTGGCCTGGTGCAATAGG 3′, probe: 5′ TGCACTGGATGCACTCTATCC CATTCTG 3′). HHV-8 DNA copy numbers in PBMCs were measured with an ORF65 Taqman assay described earlier [31]. As a control, a DNA assay was performed for β-actin using 5 µl eluate ( = 20,000 cell equivalents) as input. The β-actin reaction conditions were the same except for the primer and probe concentrations (0.3 µM forward and reverse primers and 0.2 µM Taqman probe). Following the activation of UDG (2 min, 50°C) and activation of Platinum® Taq DNA polymerase (10 min 95°C), 45 cycles (15 sec 95°C, and 1 min 60°C) were performed on an ABI 7700 Sequence Detection System (ABI, Foster City, CA, USA). The threshold cycle (Ct) for each sample of the standard curve was plotted against the input copy number. The value of Ct was determined by the first cycle number at which fluorescence was greater than the set threshold value. For accurate comparison of the samples the threshold was the same for all the experiments. Linear regression was used to determine the copy number of the experimental samples. The sialoadhesin copy numbers measured were converted to copies/10^4^ PBMC. All samples were tested in duplicate. As a control for cross contamination samples consisting of distilled water were also subjected to the isolation method and the extracts were tested with all assays. The Ct for all these “no-template” samples was always >45 cycles.

### Fluorescence-activated cell sorter (FACS) analysis

PBMCs were isolated by density centrifugation on Lymphoprep (Axis-Shield PoC AS, Norway) from three healthy donors and three treatment-naïve asymptomatic HIV-1 infected patients with HIV-1 plasma loads between 90,000 and 100,000 copies vRNA/ml.

Subsequently, 0.5–1×10^6^ cells were used for staining. All samples for FACS analysis were prepared within 2 hrs after blood was drawn. Whole PBMC fraction was gated and analysed for the expression of CD14, CD16 and CD169. Mouse anti-human monoclonal antibodies (MAbs) against the following molecules were used: PE-conjugated CD14 (Becton Dickinson, USA), FITC-conjugated CD16 (Pelicluster™ monoclonal antibodies, Sanquin, The Netherlands), and CD169 (Serotec, UK), which was detected with an APC-conjugated goat (F(ab’)_2_ anti-mouse IgG (Caltag Laboratories). The staining order was: mouse anti-CD169, then goat anti-mouse, and finally the directly labelled antibodies, with washing steps in between to minimize cross-reactivity. The samples were subsequently analysed on a FACS Caliber flow cytometer (Becton Dickinson, USA). Unstained cells and appropriate isotype-matched controls were used to determine positive CD14, CD16 and CD169 staining.

### Statistical analysis

Overall comparison was done using analysis of variance (ANOVA). If statistical significance was obtained for a variable, a post-hoc analysis was done with application of the Student's t test for group-by-group comparisons. All statistical analyses were performed with SAS software (SAS version 8.02, SAS Institute, Cary, NC, USA).
